# Threats and outbreaks of cholera in Africa amidst COVID-19 pandemic: a double burden on Africa’s health systems

**DOI:** 10.1186/s41182-021-00376-2

**Published:** 2021-11-24

**Authors:** Olivier Uwishema, Melody Okereke, Helen Onyeaka, Mohammad Mehedi Hasan, Deocles Donatus, Zebadiah Martin, Lawal Abdulwahab Oluwatomisin, Melissa Mhanna, Adesipe Olaoluwa Olumide, Jeffrey Sun, Irem Adanur

**Affiliations:** 1 Oli Health Magazine Organization, Research and Education, Kigali, Rwanda; 2 Clinton Global Initiative University, New York, USA; 3 Faculty of Medicine, Karadeniz Technical University, 61080 Trabzon, Turkey; 4 Faculty of Pharmaceutical Sciences, University of Ilorin, Kwara State, Nigeria; 5 School of Chemical Engineering, University of Birmingham, Edgbaston, Birmingham, B15 2TT UK; 6 Department of Biochemistry and Molecular Biology, Faculty of Life Science, Mawlana Bhashani Science and Technology University, Tangail, 1902 Bangladesh; 7 University of Dar es salaam Tanzania, Dar es Salaam, Tanzania; 8 Mbeya Zonal Consultant Hospital, Mbeya, Tanzania; 9 Department of Surgery, University of Ilorin Teaching Hospital, Kwara State, Nigeria; 10 Faculty of Medicine, University of Saint Joseph Beirut, Beirut, Lebanon; 11 Department of Obstetrics and Gynaecology, Olabisi Onabanjo University Teaching Hospital, Sagamu, Nigeria; 12 Faculty of Health Sciences, McMaster University, Hamilton, ON Canada

**Keywords:** Cholera, COVID-19, Africa, Health system, Double burden, Pandemic

## Abstract

Every year, about 4 million cases and 143,000 deaths due to cholera are recorded globally, of which 54% were from Africa, reported in 2016. The outbreak and spread of cholera have risen exponentially particularly in Africa. Coupled with the recent emergence of the Coronavirus Pandemic (COVID-19) in Africa, the local health systems are facing a double burden of these infectious diseases due to their cumulative impact. In this paper, we evaluate the dual impact of cholera and COVID-19 in Africa and suggest plausible interventions that can be put in place to cushion its impact.

## Background

While Severe Acute Respiratory Syndrome Coronavirus II, SARS-CoV-2 or COVID-19, is infecting and killing hundreds of African citizens, the impact of cholera, neglected due to the pandemic, is still rising very significantly. Therefore, the African continent is carrying a “double burden”. Effectively, since 1817 and until now, cholera remains a global challenge to public health and an indicator of inequity and lack of social development [1].

Cholera affects approximately 2.9 million people yearly, leading to 95,000 deaths worldwide mainly in low- and middle-income countries. In Africa alone, 40 million people live in cholera-endemic areas with a risk of frequent outbreaks [2, 3]. Throughout the centuries, African countries have been battling many outbreaks. However, the damage induced by the COVID-19 pandemic exceeds all previous and modern disease outbreaks in terms of the scope, extent, and persistence of its effects, which could undo decades of gains in public health and poverty reduction across the region [4].

Severe concerns have been pointed out regarding the flare-up of cholera during the COVID-19 pandemic. There has been an increase in reported cholera cases in endemic areas compared to previous years. The COVID-19 pandemic has disturbed the diagnosis and treatment services, and vaccination campaigns, including cholera vaccines, therefore, putting millions of lives at risk of vaccine-preventable disease [5, 6]. Furthermore, the COVID-19 pandemic has caused a lack of access to humanitarian aid and also an increased pressure on health systems in cholera-endemic countries, making it impossible to handle two or more simultaneous outbreaks due to lack of capacity in personnel and supplies [7]. Additionally, the World Health Organization (WHO) and United Nations Children’s Fund (UNICEF) have experienced challenges in dealing with the recent cholera outbreaks in many African Countries amidst the COVID-19 pandemic as it has added an extra burden on the already existing, limited resources [8, 9].

However, the pandemic has helped in spreading awareness on essential infection control measures: it has highlighted the importance of personal hygiene practices, the importance of vaccines in the protection against infectious disease and the role of prediction, preparedness, and early response that should be deployed to slow the spread of cholera [2].

With the COVID-19 outbreak, the increased threat of cholera should not be overlooked, especially that all regional health organizations are weighed down by this new pandemic. At this stage, international and humanitarian bodies may be essential to equip and prepare the African institutions to face and minimize the impact of even any potential pandemic in the future.

## The situation of fighting cholera before COVID 19 pandemic in Africa

In March 2020, WHO declared COVID 19 arising from Wuhan province in China as a global pandemic and as a global threat [10]. This devastating news affected almost every sector including shifting attention from the existing fatal diseases that have claimed many people’s lives far more than the COVID 19 [11].

Cholera has caused havoc for centuries affecting almost every continent, currently with a higher incidence in developing countries, being associated with poverty [12]. As cholera remains a significant global public health concern, its occurrence is linked to insufficient access to safe water and proper sanitation. Oral cholera vaccine (OCV) has been recommended as an additional public health tool along with the safe Water, Sanitation and Hygiene (WASH) technique as a measure to eliminate the disease [13]. Before the emergence of vaccine, the WASH strategy was widely adopted in the fight against the disease in African countries [13].

OCV seems to be cost-effective as part of the prevention and control measure of cholera when targeted at the population with a high risk of cholera and poor access to healthcare facilities and has been employed in some countries of Africa, such as Malawi, Mozambique, and Ethiopia [14].

In 2011, WHO established a global oral cholera vaccine stockpile aiming at the rapid response to cholera outbreaks. From 2013 to 2017, over 25 million doses have been requested from the cholera vaccine stockpile, but only 51% were shipped to countries in need some African countries such as Malawi and Mozambique [14, 15].

## Burden of cholera during the COVID 19 outbreak in Africa

Cholera outbreak remains a major public health concern since its first outbreak [2, 3, 16]. High-risk areas are peri-urban slums, concentration camps for displaced populations or refugees where the minimum requirement for clean and safe water and sanitation system requirements is not always met [1]. Disruption of humanitarian services to high-risk areas poses great setbacks in the fight against the cholera outbreak due to poor surveillance of the outbreak.

Due to lack of effective surveillance and weakening of healthcare system as efforts are being directed towards fight against COVID-19, data regarding cholera outbreaks are limited and uncertain in most African countries [1]. However, recently in the past 2 years, cholera outbreaks have been reported in Ethiopia and Sudan, both cholera-endemic regions coinciding with COVID-19 era [17]. This has been evidenced in reports from Ethiopia, where disruption of response to cholera response during COVID-19 has led to approximately 15,000 cases and 250 deaths [17]. Conflicts also contributed to poor response to cholera outbreak, especially in the Tigray region, which caused Ethiopians to flee to Sudan, staying in refugee camps with disrupted WASH services. Disruption of WASH services coinciding with restrictions of humanitarian services, and the economic strains developed during COVID-19 are a great hindrance to campaigns against cholera outbreak, thus worsening the burden.

The last cholera outbreak in Somalia dates back to 2017 following a flood that occurred in Jubba basin and Shabelle River. The outbreak was contained in five out of six regions. However, as of April 2020, an increased number of cases was reported following a flood caused by heavy rainfalls, with the cumulative attack rate increasing to 26 persons per 100,000 by 2021 [8]. These findings suggest the role of naturally occurring events such as a flood in spreading the outbreak, but on the other hand, this presents critical failure of healthcare system response to cholera outbreak which occurred mainly during COVID-19 era. In addition to cholera, some African countries have faced other infectious diseases and viral outbreaks such as bird flu, malaria, Ebola, measles, dengue, plague and Lassa fever [18–25].

## Efforts and challenges to cholera response amidst COVID-19 in Africa

The widespread cholera epidemics in Africa have been linked to overpopulation, water, sanitation issues, and penury [26]. The prevention of cholera is focused on improving sanitation and hygiene standards. The World Health Organization (WHO) also endorses the oral cholera vaccines usage as a temporary preventative measure. There are several hurdles such as fragile socio-economic infrastructure, lack of adequate resources, logistics impeding widespread and pragmatic use of cholera vaccines in Africa, and vaccination rates remain low [27]. Cholera prevention and control strategy was adopted by 47 African countries prior to the COVID-19 pandemic. The African countries have agreed to reduce cholera incidence by implementing evidence-based steps, strengthening treatment methods, strengthening border monitoring, and encouraging oral cholera vaccines use while expanding water supply investments [28]. The pandemic, as expected, had an adverse influence on many schemes that could be implemented, such as vaccine advertising efforts, supplies, launching surveillance system, hygiene programs that were victimized due to lockdown initiatives, quarantine and social distance to help deter spreading the disease. Therefore, national control and prevention strategies should be implemented, providing effective and affordable diagnostics and treatment for cholera and COVID-19, enhancing laboratory diagnostics, clinical management, environmental control and research. The community’s involvement is highly recommended, and the development of an effective prevention strategy would be the key pillars to mitigate cholera incidence, especially during the challenging times of the COVID-19 pandemic.

## Recommendations

**Fig. 1:**
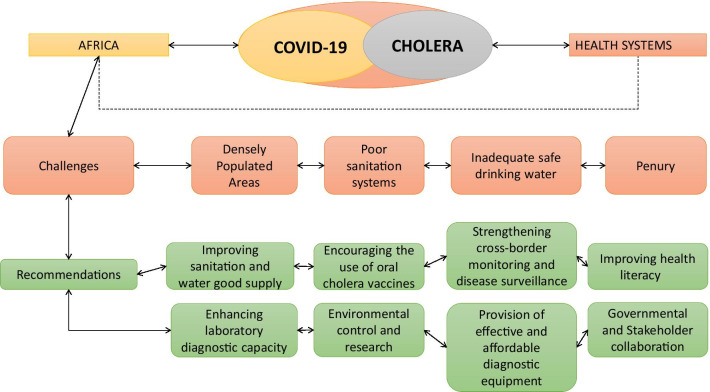
A summary of challenges, priorities, and recommendations for the restriction of cholera pathogen amid the COVID-19 pandemic

The emergence of COVID-19 has disrupted the hard-won years of progress against cholera in Africa, but despite the paradigm shift in focus on COVID-19, it is critical that cholera must not become more forgotten. While the focus is on COVID-19, efforts such as improving water supply investment and sanitation, encouraging the use of oral cholera vaccines, strengthening cross-border surveillance, government, and stakeholder collaboration are necessary to counter the burden of cholera. A summary of challenges and recommendations is provided in the figure below (Fig. 1).

## Conclusion

Africa is a high-risk area for the spread of cholera due to poor sanitation systems, lack of good drinking water, and densely populated areas. Disruption of healthcare services in high-risk areas due to COVID-19 therefore poses a great challenge in the fight against cholera. As priority is now being shifted towards COVID-19 containment, data regarding cholera outbreaks are limited and unavailable in most African countries resulting in a lapse in contact tracing and the management of cholera patients as they are left undiagnosed and untreated. To win the fight against cholera and COVID-19 simultaneously, a ‘whole of Africa’ integrated approach involving collective advocacies, cross-border surveillance and information sharing, and stakeholder collaboration would be crucial for progress. This will ensure that no country is left behind because no one is safe until everyone is safe.

## Data Availability

Not applicable.

## Abbreviations

COVID-19: Coronavirus Disease 2019
WHO: World Health Organization
UNICEF: United Nations Children’s Emergency Fund
OCV: Oral cholera vaccine
WASH: Water, Sanitation and Hygiene
